# Bis{[μ-bis­(diphenyl­arsino)methane-1:2κ^2^ *As*:*As*′]nona­carbonyl-1κ^3^ *C*,2κ^3^ *C*,3κ^3^ *C*-[tris­(4-methoxy­phen­yl)arsine-3κ*As*]-*triangulo*-triruthenium(0)} dichloro­methane solvate

**DOI:** 10.1107/S160053680905315X

**Published:** 2010-01-16

**Authors:** Omar bin Shawkataly, Imthyaz Ahmed Khan, Chin Sing Yeap, Hoong-Kun Fun

**Affiliations:** a Chemical Sciences Programme, School of Distance Education, Universiti Sains Malaysia, 11800 USM, Penang, Malaysia; b X-ray Crystallography Unit, School of Physics, Universiti Sains Malaysia, 11800 USM, Penang, Malaysia

## Abstract

The asymmetric unit of the title *triangulo*-triruthenium compound, 2[Ru_3_(C_25_H_22_As_2_)(C_21_H_21_AsO_3_)(CO)_9_]·CH_2_Cl_2_, contains one *triangulo*-triruthenium complex mol­ecule and one half of the dichloro­methane solvent. The dichloro­methane solvent lies across a crystallographic inversion center leading to the mol­ecule being disordered over two positions of equal occupancy. The bis­(diphenyl­arsino)methane ligand bridges an Ru—Ru bond and the monodentate arsine ligand bonds to the third Ru atom. Both the arsine ligands are equatorial with respect to the Ru_3_ triangle. In addition, each Ru atom carries one equatorial and two axial terminal carbonyl ligands. The trimethoxy­phenyl­arsino benzene rings make dihedral angles of 83.01 (8), 65.81 (8) and 76.20 (8)° with each other. The dihedral angles between the two benzene rings are 82.69 (9) and 78.83 (9)° for the two diphenyl­arsino groups. In the crystal packing, the mol­ecules are stacked along the *a* axis and weak inter­molecular C—H⋯π inter­actions stabilize the crystal structure.

## Related literature

For general background to *triangulo*-triruthenium derivatives, see: Bruce *et al.* (1985 ▶, 1988*a* ▶,*b* ▶). For related structures, see: Shawkataly *et al.* (1998 ▶, 2004 ▶, 2009 ▶, 2010 ▶). For the synthesis of tris­(4-methoxy­phen­yl)arsine, see: Blicke & Cataline (1938 ▶). For the synthesis of μ-bis­(diphenyl­arsino)methane­deca­carbonyl­tri­ruth­enium(0), see: Bruce *et al.* (1983 ▶). For the stability of the temperature controller used for the data collection, see: Cosier & Glazer (1986 ▶).
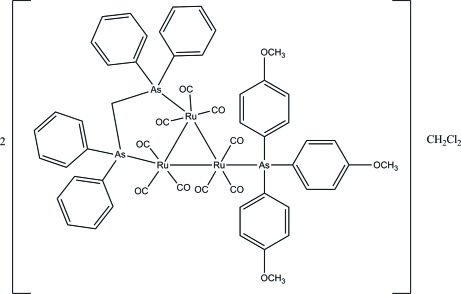


## Experimental

### 

#### Crystal data

2[Ru_3_(C_25_H_22_As_2_)(C_21_H_21_AsO_3_)(CO)_9_]·CH_2_Cl_2_
*M* *_r_* = 2932.65
Triclinic, 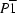

*a* = 10.7669 (1) Å
*b* = 12.8159 (2) Å
*c* = 20.7167 (2) Å
α = 95.997 (1)°
β = 101.259 (1)°
γ = 103.451 (1)°
*V* = 2692.85 (6) Å^3^
*Z* = 1
Mo *K*α radiation
μ = 2.77 mm^−1^
*T* = 100 K
0.32 × 0.25 × 0.18 mm

#### Data collection

Bruker SMART APEXII CCD area-detector diffractometer
Absorption correction: multi-scan (*SADABS*; Bruker, 2005 ▶) *T*_min_ = 0.472, *T*_max_ = 0.631
90976 measured reflections
19440 independent reflections
17122 reflections with *I* > 2σ(*I*)
*R*_int_ = 0.025

#### Refinement

*R*[*F* ^2^ > 2σ(*F* ^2^)] = 0.021
*wR*(*F* ^2^) = 0.052
*S* = 1.01
19440 reflections
679 parameters
H-atom parameters constrained
Δρ_max_ = 0.73 e Å^−3^
Δρ_min_ = −1.73 e Å^−3^

### 

Data collection: *APEX2* (Bruker, 2005 ▶); cell refinement: *SAINT* (Bruker, 2005 ▶); data reduction: *SAINT*; program(s) used to solve structure: *SHELXTL* (Sheldrick, 2008 ▶); program(s) used to refine structure: *SHELXTL*; molecular graphics: *SHELXTL*; software used to prepare material for publication: *SHELXTL* and *PLATON* (Spek, 2009 ▶).

## Supplementary Material

Crystal structure: contains datablocks global, I. DOI: 10.1107/S160053680905315X/tk2594sup1.cif

Structure factors: contains datablocks I. DOI: 10.1107/S160053680905315X/tk2594Isup2.hkl

Additional supplementary materials:  crystallographic information; 3D view; checkCIF report

## Figures and Tables

**Table 1:** Hydrogen-bond geometry (Å, °)

*D*—H⋯*A*	*D*—H	H⋯*A*	*D*⋯*A*	*D*—H⋯*A*
C17—H17*A*⋯*Cg*1^i^	0.93	2.92	3.813 (2)	161
C22—H22*A*⋯*Cg*2^ii^	0.93	2.89	3.5863 (19)	133
C54—H54*C*⋯*Cg*3^iii^	0.96	2.87	3.710 (2)	147

Symmetry codes: (i) 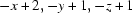; (ii) 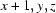; (iii) 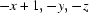. *Cg*1 *Cg*2 and *Cg*3 are the centroids of the C38–C43, C26–C31 and C32–C37 benzene rings, respectively.

**Fig. 1.**
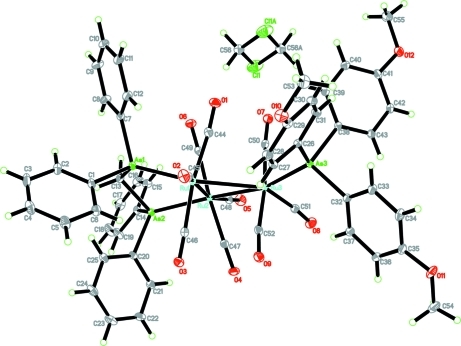
The molecular structure of the title compound with 30% probability ellipsoids for non-H atoms. Atoms with suffix A are generated by the symmetry operation (1 - x, 1 - y, 1 - z).

**Fig. 2.**
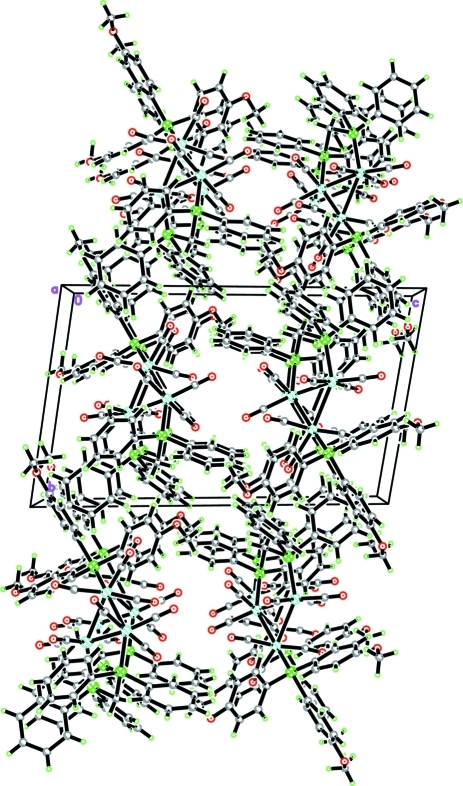
The crystal packing of the title compound, viewed down the a axis, showing the molecules stacked along a axis. The solvent molecules have been omitted for clarity.

## supplementary crystallographic information

### Comment

*Triangulo*-triruthenium clusters are known for their interesting structural variations and related catalytic activity. A large number of substituted derivatives, Ru_3_(CO)_12-n_*L*_n_ (*L*=15 group ligand) have been reported (Bruce *et al.*, 1985, 1988*a*,*b*). As part of our study on the substitution of transition metal-carbonyl clusters with mixed-ligand complexes, we have published several structures of *triangulo*-triruthenium-carbonyl clusters containing mixed P/As and P/Sb ligands (Shawkataly *et al.*, 1998, 2004, 2009,2010). Herein, we report the synthesis and structure of title compound.

The asymmetric unit consists of one molecule of the *triangulo*-triruthenium complex and half a molecule of dichloromethane solvent (Fig. 1). The dichloromethane solvent lies across a crystallographic inversion center leading to the molecule being disordered over two positions of equal occupancy. The bond lengths and angles of title compound are comparable to those found in related structure (Shawkataly *et al.*, 2009,2010). The bis(diphenylarsino)methane ligand bridges the Ru1—Ru2 bond and the monodentate arsine ligand bonds to the Ru3 atom. Both the arsine ligands are equatorial with respect to the Ru_3_ triangle. Additionally, each Ru atom carries one equatorial and two axial terminal carbonyl ligands. The trimethoxyphenylarsino benzene rings make dihedral angles (C26–C31/C32–C37, C26–C31/C38–C43 and C32–C37/C38–C43) of 83.01 (8), 65.81 (8) and 76.20 (8)° with each other, respectively. The dihedral angles between the two benzene rings (C1–C6/C7–C12 and C14–C19/C20–C25) are 82.69 (9) and 78.83 (9)° for the two diphenylarsino groups, respectively.

In the crystal packing (Fig. 2), the molecules are stacked along *a* axis and weak intermolecular C—H···π interactions stabilize the crystal structure (Table 1).

### Experimental

The reactions were conducted under a atmosphere of high purity nitrogen using standard Schlenk techniques and hexane-dried over sodium metal. Tris(4-methoxyphenyl)arsine (Blicke *et al.*, 1938) and bis(diphenylarsino)methanedecacarbonyltriruthenium(0) (Bruce *et al.*, 1983) were prepared by reported procedures. The title compound was obtained by refluxing equimolar quantities of Ru_3_(CO)_10_(µ-Ph_2_AsCH_2_AsPh_2_) (105.5 mg, 0.1 mmol) and tris(4-methoxyphenyl)arsine (39.63 mg, 0.1 mmol) in hexane under nitrogen atmosphere. Crystals suitable for X-ray diffraction were grown by slow solvent / solvent diffusion of CH_3_OH into CHCl_3_.

### Refinement

All hydrogen atoms were positioned geometrically and refined using a riding model with C—H = 0.93–0.97 Å and *U*_iso_(H) = 1.2 or 1.5 *U*_eq_(C). A rotating group model was applied for the methyl groups. The dichloromethane solvent lies across a crystallographic inversion center leading to the molecule being disordered over two positions of equal occupancy. The maximum and minimum residual electron density peaks of 0.73 and -1.73 eÅ^-3^, respectively, were located 0.84 Å and 0.56 Å from the Cl1 atom.

### Crystal data

**Table:**

2[Ru_3_(C_25_H_22_As_2_)(C_21_H_21_As)(CO)_9_]·CH_2_Cl_2_	*Z* = 1
*M**_r_* = 2932.65	*F*(000) = 1442
Triclinic, *P*1	*D*_x_ = 1.808 Mg m^−^^3^
Hall symbol: -P 1	Mo *K*α radiation, λ = 0.71073 Å
*a* = 10.7669 (1) Å	Cell parameters from 9265 reflections
*b* = 12.8159 (2) Å	θ = 2.4–35.3°
*c* = 20.7167 (2) Å	µ = 2.77 mm^−^^1^
α = 95.997 (1)°	*T* = 100 K
β = 101.259 (1)°	Block, purple
γ = 103.451 (1)°	0.32 × 0.25 × 0.18 mm
*V* = 2692.85 (6) Å^3^	

### Data collection

**Table:**

Bruker SMART APEXII CCD area-detector diffractometer	19440 independent reflections
Radiation source: fine-focus sealed tube	17122 reflections with *I* > 2σ(*I*)
graphite	*R*_int_ = 0.025
φ and ω scans	θ_max_ = 32.5°, θ_min_ = 2.0°
Absorption correction: multi-scan (*SADABS*; Bruker, 2005)	*h* = −16→16
*T*_min_ = 0.472, *T*_max_ = 0.631	*k* = −19→19
90976 measured reflections	*l* = −31→31

### Refinement

**Table:**

Refinement on *F*^2^	Primary atom site location: structure-invariant direct methods
Least-squares matrix: full	Secondary atom site location: difference Fourier map
*R*[*F*^2^ > 2σ(*F*^2^)] = 0.021	Hydrogen site location: inferred from neighbouring sites
*wR*(*F*^2^) = 0.052	H-atom parameters constrained
*S* = 1.01	*w* = 1/[σ^2^(*F*_o_^2^) + (0.0223*P*)^2^ + 2.0523*P*] where *P* = (*F*_o_^2^ + 2*F*_c_^2^)/3
19440 reflections	(Δ/σ)_max_ = 0.002
679 parameters	Δρ_max_ = 0.73 e Å^−^^3^
0 restraints	Δρ_min_ = −1.73 e Å^−^^3^

### Special details

**Table:**

Experimental. The crystal was placed in the cold stream of an Oxford Cyrosystems Cobra open-flow nitrogen cryostat (Cosier & Glazer, 1986) operating at 100.0 (1) K.
Geometry. All e.s.d.'s (except the e.s.d. in the dihedral angle between two l.s. planes) are estimated using the full covariance matrix. The cell e.s.d.'s are taken into account individually in the estimation of e.s.d.'s in distances, angles and torsion angles; correlations between e.s.d.'s in cell parameters are only used when they are defined by crystal symmetry. An approximate (isotropic) treatment of cell e.s.d.'s is used for estimating e.s.d.'s involving l.s. planes.
Refinement. Refinement of *F*^2^ against ALL reflections. The weighted *R*-factor *wR* and goodness of fit *S* are based on *F*^2^, conventional *R*-factors *R* are based on *F*, with *F* set to zero for negative *F*^2^. The threshold expression of *F*^2^ > σ(*F*^2^) is used only for calculating *R*-factors(gt) *etc*. and is not relevant to the choice of reflections for refinement. *R*-factors based on *F*^2^ are statistically about twice as large as those based on *F*, and *R*- factors based on ALL data will be even larger.

### Fractional atomic coordinates and isotropic or equivalent isotropic displacement parameters (Å^2^)

**Table:**

	*x*	*y*	*z*	*U*_iso_*/*U*_eq_	Occ. (<1)
Ru1	0.748217 (11)	0.570966 (9)	0.225464 (6)	0.01353 (2)	
Ru2	0.923559 (11)	0.508430 (9)	0.327473 (5)	0.01269 (2)	
Ru3	0.691165 (11)	0.354235 (9)	0.257861 (6)	0.01359 (2)	
As1	0.850968 (14)	0.763617 (12)	0.262954 (7)	0.01454 (3)	
As2	1.092347 (14)	0.673703 (12)	0.332126 (7)	0.01360 (3)	
As3	0.464318 (14)	0.251824 (12)	0.208703 (7)	0.01442 (3)	
O1	0.53976 (12)	0.57997 (11)	0.30693 (7)	0.0276 (3)	
O2	0.57004 (12)	0.59551 (11)	0.09838 (6)	0.0274 (3)	
O3	0.95014 (12)	0.53629 (10)	0.14399 (6)	0.0227 (2)	
O4	1.02873 (12)	0.36228 (10)	0.23643 (6)	0.0255 (2)	
O5	1.03705 (14)	0.40670 (12)	0.44375 (7)	0.0312 (3)	
O6	0.80068 (12)	0.63916 (10)	0.41856 (6)	0.0243 (2)	
O7	0.60368 (13)	0.40703 (10)	0.38771 (6)	0.0254 (2)	
O8	0.80348 (14)	0.17286 (11)	0.30517 (7)	0.0299 (3)	
O9	0.76267 (13)	0.31181 (11)	0.12285 (6)	0.0281 (3)	
O10	0.09041 (12)	0.38956 (11)	−0.00395 (6)	0.0268 (3)	
O11	0.38176 (14)	−0.16804 (10)	0.01920 (6)	0.0294 (3)	
O12	0.15856 (13)	0.08073 (10)	0.41159 (6)	0.0268 (3)	
C1	0.89112 (15)	0.85813 (13)	0.19751 (8)	0.0192 (3)	
C2	0.92364 (18)	0.97072 (14)	0.21647 (10)	0.0271 (3)	
H2A	0.9295	1.0002	0.2603	0.033*	
C3	0.9473 (2)	1.03876 (15)	0.16951 (11)	0.0341 (4)	
H3A	0.9688	1.1137	0.1820	0.041*	
C4	0.9387 (2)	0.99484 (17)	0.10422 (10)	0.0345 (4)	
H4A	0.9549	1.0405	0.0731	0.041*	
C5	0.9062 (2)	0.88321 (16)	0.08523 (10)	0.0321 (4)	
H5A	0.9003	0.8542	0.0413	0.038*	
C6	0.88232 (17)	0.81427 (14)	0.13187 (8)	0.0243 (3)	
H6A	0.8606	0.7393	0.1191	0.029*	
C7	0.75040 (15)	0.84464 (12)	0.30631 (8)	0.0180 (3)	
C8	0.80015 (17)	0.91164 (14)	0.36772 (9)	0.0246 (3)	
H8A	0.8858	0.9186	0.3906	0.029*	
C9	0.72189 (19)	0.96824 (16)	0.39495 (10)	0.0296 (4)	
H9A	0.7549	1.0124	0.4362	0.036*	
C10	0.59524 (18)	0.95882 (15)	0.36063 (11)	0.0300 (4)	
H10A	0.5431	0.9968	0.3788	0.036*	
C11	0.54584 (17)	0.89312 (15)	0.29933 (11)	0.0300 (4)	
H11A	0.4607	0.8874	0.2762	0.036*	
C12	0.62267 (16)	0.83559 (14)	0.27205 (9)	0.0245 (3)	
H12A	0.5888	0.7910	0.2309	0.029*	
C13	1.01597 (14)	0.79804 (12)	0.33037 (8)	0.0175 (3)	
H13A	1.0774	0.8594	0.3206	0.021*	
H13B	1.0000	0.8183	0.3738	0.021*	
C14	1.22911 (15)	0.72635 (12)	0.41276 (7)	0.0174 (3)	
C15	1.19085 (17)	0.72494 (14)	0.47329 (8)	0.0230 (3)	
H15A	1.1029	0.6990	0.4738	0.028*	
C16	1.28408 (19)	0.76219 (15)	0.53279 (8)	0.0281 (4)	
H16A	1.2584	0.7620	0.5731	0.034*	
C17	1.41483 (19)	0.79953 (16)	0.53228 (9)	0.0314 (4)	
H17A	1.4773	0.8230	0.5723	0.038*	
C18	1.45289 (19)	0.80199 (18)	0.47264 (10)	0.0360 (4)	
H18A	1.5409	0.8284	0.4725	0.043*	
C19	1.36012 (17)	0.76501 (16)	0.41236 (9)	0.0284 (4)	
H19A	1.3862	0.7663	0.3722	0.034*	
C20	1.18625 (14)	0.68358 (13)	0.26161 (7)	0.0171 (3)	
C21	1.24764 (15)	0.60231 (13)	0.24738 (8)	0.0204 (3)	
H21A	1.2484	0.5479	0.2737	0.024*	
C22	1.30791 (16)	0.60245 (15)	0.19368 (8)	0.0245 (3)	
H22A	1.3482	0.5478	0.1840	0.029*	
C23	1.30788 (17)	0.68386 (17)	0.15467 (8)	0.0278 (4)	
H23A	1.3481	0.6838	0.1188	0.033*	
C24	1.24798 (17)	0.76539 (16)	0.16906 (9)	0.0278 (4)	
H24A	1.2486	0.8202	0.1429	0.033*	
C25	1.18693 (15)	0.76580 (14)	0.22238 (8)	0.0211 (3)	
H25A	1.1467	0.8206	0.2318	0.025*	
C26	0.34264 (14)	0.31040 (12)	0.15043 (7)	0.0162 (3)	
C27	0.38765 (15)	0.36185 (12)	0.10008 (8)	0.0188 (3)	
H27A	0.4766	0.3785	0.1005	0.023*	
C28	0.30132 (17)	0.38827 (14)	0.04953 (8)	0.0218 (3)	
H28A	0.3323	0.4222	0.0161	0.026*	
C29	0.16790 (16)	0.36393 (13)	0.04880 (8)	0.0198 (3)	
C30	0.12182 (16)	0.31616 (13)	0.10001 (8)	0.0204 (3)	
H30A	0.0334	0.3024	0.1007	0.024*	
C31	0.20998 (15)	0.28927 (13)	0.15017 (8)	0.0191 (3)	
H31A	0.1795	0.2566	0.1841	0.023*	
C32	0.44377 (15)	0.11738 (12)	0.14984 (7)	0.0172 (3)	
C33	0.32017 (16)	0.06402 (13)	0.10922 (8)	0.0212 (3)	
H33A	0.2486	0.0921	0.1116	0.025*	
C34	0.30393 (18)	−0.02992 (13)	0.06566 (9)	0.0248 (3)	
H34A	0.2217	−0.0646	0.0387	0.030*	
C35	0.41039 (18)	−0.07291 (13)	0.06199 (8)	0.0218 (3)	
C36	0.53369 (17)	−0.02054 (13)	0.10135 (8)	0.0229 (3)	
H36A	0.6053	−0.0484	0.0987	0.027*	
C37	0.54874 (16)	0.07457 (13)	0.14493 (8)	0.0215 (3)	
H37A	0.6313	0.1099	0.1713	0.026*	
C38	0.36401 (14)	0.20005 (12)	0.27262 (7)	0.0162 (3)	
C39	0.31161 (16)	0.26948 (13)	0.30952 (8)	0.0206 (3)	
H39A	0.3242	0.3416	0.3030	0.025*	
C40	0.24104 (17)	0.23255 (13)	0.35581 (8)	0.0222 (3)	
H40A	0.2043	0.2790	0.3790	0.027*	
C41	0.22564 (15)	0.12585 (13)	0.36729 (8)	0.0195 (3)	
C42	0.28230 (15)	0.05689 (13)	0.33270 (8)	0.0195 (3)	
H42A	0.2754	−0.0136	0.3417	0.023*	
C43	0.34871 (15)	0.09347 (12)	0.28500 (8)	0.0180 (3)	
H43A	0.3834	0.0464	0.2610	0.022*	
C44	0.61826 (15)	0.57114 (13)	0.27824 (8)	0.0195 (3)	
C45	0.63364 (15)	0.58388 (13)	0.14722 (8)	0.0191 (3)	
C46	0.87797 (15)	0.54744 (12)	0.17636 (7)	0.0184 (3)	
C47	0.98557 (14)	0.41806 (13)	0.26738 (8)	0.0180 (3)	
C48	0.99577 (15)	0.44521 (13)	0.39973 (8)	0.0193 (3)	
C49	0.84055 (14)	0.58853 (12)	0.38269 (7)	0.0173 (3)	
C50	0.64039 (15)	0.39241 (12)	0.34029 (8)	0.0191 (3)	
C51	0.75530 (16)	0.23904 (13)	0.28797 (8)	0.0200 (3)	
C52	0.73926 (15)	0.33497 (13)	0.17343 (8)	0.0198 (3)	
C53	−0.04799 (18)	0.36350 (16)	−0.00756 (9)	0.0295 (4)	
H53A	−0.0915	0.3866	−0.0464	0.044*	
H53B	−0.0643	0.4001	0.0316	0.044*	
H53C	−0.0807	0.2864	−0.0102	0.044*	
C54	0.4875 (2)	−0.21280 (16)	0.01151 (10)	0.0334 (4)	
H54A	0.4543	−0.2822	−0.0167	0.050*	
H54B	0.5335	−0.2216	0.0544	0.050*	
H54C	0.5463	−0.1649	−0.0084	0.050*	
C55	0.0980 (2)	0.14896 (17)	0.44722 (10)	0.0350 (4)	
H55A	0.0518	0.1084	0.4756	0.052*	
H55B	0.0375	0.1742	0.4161	0.052*	
H55C	0.1641	0.2101	0.4738	0.052*	
Cl1	0.62312 (7)	0.49780 (6)	0.54517 (3)	0.05626 (16)	
C56	0.5351 (4)	0.5640 (3)	0.4957 (2)	0.0337 (8)	0.50
H56A	0.5828	0.5856	0.4626	0.040*	0.50
H56B	0.5342	0.6294	0.5225	0.040*	0.50

### Atomic displacement parameters (Å^2^)

**Table:**

	*U*^11^	*U*^22^	*U*^33^	*U*^12^	*U*^13^	*U*^23^
Ru1	0.01293 (5)	0.01240 (5)	0.01399 (5)	0.00233 (4)	0.00091 (4)	0.00267 (4)
Ru2	0.01146 (5)	0.01378 (5)	0.01296 (5)	0.00336 (4)	0.00268 (3)	0.00265 (4)
Ru3	0.01337 (5)	0.01205 (5)	0.01495 (5)	0.00242 (4)	0.00324 (4)	0.00227 (4)
As1	0.01342 (6)	0.01264 (6)	0.01655 (7)	0.00272 (5)	0.00162 (5)	0.00260 (5)
As2	0.01158 (6)	0.01491 (6)	0.01367 (6)	0.00274 (5)	0.00211 (5)	0.00245 (5)
As3	0.01369 (6)	0.01293 (6)	0.01654 (7)	0.00316 (5)	0.00390 (5)	0.00164 (5)
O1	0.0234 (6)	0.0346 (7)	0.0321 (7)	0.0141 (5)	0.0114 (5)	0.0130 (5)
O2	0.0247 (6)	0.0316 (7)	0.0235 (6)	0.0055 (5)	−0.0014 (5)	0.0109 (5)
O3	0.0221 (5)	0.0258 (6)	0.0201 (5)	0.0050 (4)	0.0060 (4)	0.0041 (4)
O4	0.0235 (6)	0.0281 (6)	0.0275 (6)	0.0106 (5)	0.0093 (5)	−0.0001 (5)
O5	0.0327 (7)	0.0376 (7)	0.0278 (6)	0.0150 (6)	0.0047 (5)	0.0159 (6)
O6	0.0228 (6)	0.0276 (6)	0.0225 (6)	0.0088 (5)	0.0061 (4)	−0.0021 (5)
O7	0.0303 (6)	0.0244 (6)	0.0229 (6)	0.0057 (5)	0.0108 (5)	0.0035 (5)
O8	0.0374 (7)	0.0268 (6)	0.0316 (7)	0.0172 (6)	0.0086 (5)	0.0103 (5)
O9	0.0308 (7)	0.0271 (6)	0.0225 (6)	−0.0012 (5)	0.0106 (5)	−0.0023 (5)
O10	0.0256 (6)	0.0345 (7)	0.0203 (6)	0.0103 (5)	0.0006 (4)	0.0072 (5)
O11	0.0428 (8)	0.0188 (6)	0.0239 (6)	0.0087 (5)	0.0048 (5)	−0.0046 (5)
O12	0.0318 (7)	0.0243 (6)	0.0263 (6)	0.0031 (5)	0.0150 (5)	0.0061 (5)
C1	0.0158 (6)	0.0177 (7)	0.0235 (7)	0.0025 (5)	0.0033 (5)	0.0072 (5)
C2	0.0295 (9)	0.0183 (7)	0.0322 (9)	0.0021 (6)	0.0073 (7)	0.0064 (6)
C3	0.0380 (10)	0.0196 (8)	0.0442 (11)	0.0012 (7)	0.0114 (8)	0.0132 (8)
C4	0.0358 (10)	0.0317 (10)	0.0367 (10)	0.0030 (8)	0.0095 (8)	0.0189 (8)
C5	0.0375 (10)	0.0328 (9)	0.0252 (8)	0.0047 (8)	0.0072 (7)	0.0114 (7)
C6	0.0274 (8)	0.0214 (7)	0.0233 (8)	0.0042 (6)	0.0048 (6)	0.0065 (6)
C7	0.0169 (6)	0.0143 (6)	0.0240 (7)	0.0051 (5)	0.0050 (5)	0.0048 (5)
C8	0.0224 (7)	0.0255 (8)	0.0258 (8)	0.0112 (6)	0.0017 (6)	−0.0004 (6)
C9	0.0305 (9)	0.0294 (9)	0.0307 (9)	0.0142 (7)	0.0067 (7)	−0.0019 (7)
C10	0.0249 (8)	0.0229 (8)	0.0472 (11)	0.0118 (7)	0.0132 (8)	0.0055 (7)
C11	0.0181 (7)	0.0226 (8)	0.0485 (11)	0.0078 (6)	0.0035 (7)	0.0034 (7)
C12	0.0183 (7)	0.0205 (7)	0.0322 (9)	0.0056 (6)	0.0009 (6)	0.0006 (6)
C13	0.0147 (6)	0.0159 (6)	0.0197 (7)	0.0035 (5)	0.0006 (5)	0.0007 (5)
C14	0.0170 (6)	0.0171 (6)	0.0155 (6)	0.0022 (5)	0.0002 (5)	0.0026 (5)
C15	0.0219 (7)	0.0282 (8)	0.0188 (7)	0.0084 (6)	0.0028 (6)	0.0020 (6)
C16	0.0344 (9)	0.0320 (9)	0.0165 (7)	0.0123 (7)	0.0002 (6)	−0.0002 (6)
C17	0.0315 (9)	0.0297 (9)	0.0232 (8)	0.0036 (7)	−0.0087 (7)	−0.0015 (7)
C18	0.0201 (8)	0.0460 (12)	0.0313 (9)	−0.0044 (8)	−0.0041 (7)	0.0080 (8)
C19	0.0177 (7)	0.0407 (10)	0.0216 (8)	−0.0009 (7)	0.0007 (6)	0.0078 (7)
C20	0.0135 (6)	0.0206 (7)	0.0157 (6)	0.0019 (5)	0.0032 (5)	0.0026 (5)
C21	0.0172 (7)	0.0237 (7)	0.0214 (7)	0.0059 (6)	0.0058 (5)	0.0047 (6)
C22	0.0177 (7)	0.0325 (9)	0.0221 (7)	0.0054 (6)	0.0058 (6)	−0.0013 (6)
C23	0.0188 (7)	0.0452 (11)	0.0180 (7)	0.0040 (7)	0.0058 (6)	0.0052 (7)
C24	0.0234 (8)	0.0380 (10)	0.0228 (8)	0.0043 (7)	0.0065 (6)	0.0143 (7)
C25	0.0172 (7)	0.0246 (8)	0.0209 (7)	0.0031 (6)	0.0038 (5)	0.0070 (6)
C26	0.0170 (6)	0.0151 (6)	0.0166 (6)	0.0044 (5)	0.0041 (5)	0.0019 (5)
C27	0.0181 (7)	0.0183 (7)	0.0201 (7)	0.0037 (5)	0.0065 (5)	0.0022 (5)
C28	0.0249 (8)	0.0239 (7)	0.0177 (7)	0.0057 (6)	0.0072 (6)	0.0046 (6)
C29	0.0225 (7)	0.0201 (7)	0.0164 (6)	0.0073 (6)	0.0018 (5)	0.0017 (5)
C30	0.0175 (7)	0.0232 (7)	0.0213 (7)	0.0069 (6)	0.0042 (5)	0.0038 (6)
C31	0.0185 (7)	0.0204 (7)	0.0204 (7)	0.0064 (5)	0.0065 (5)	0.0060 (5)
C32	0.0188 (6)	0.0150 (6)	0.0174 (6)	0.0036 (5)	0.0049 (5)	0.0016 (5)
C33	0.0202 (7)	0.0173 (7)	0.0240 (7)	0.0049 (5)	0.0011 (6)	0.0009 (6)
C34	0.0260 (8)	0.0183 (7)	0.0250 (8)	0.0038 (6)	−0.0021 (6)	0.0008 (6)
C35	0.0322 (8)	0.0149 (6)	0.0182 (7)	0.0047 (6)	0.0073 (6)	0.0020 (5)
C36	0.0257 (8)	0.0190 (7)	0.0256 (8)	0.0066 (6)	0.0101 (6)	0.0014 (6)
C37	0.0193 (7)	0.0190 (7)	0.0251 (7)	0.0042 (5)	0.0059 (6)	−0.0008 (6)
C38	0.0151 (6)	0.0154 (6)	0.0172 (6)	0.0026 (5)	0.0036 (5)	0.0021 (5)
C39	0.0238 (7)	0.0158 (6)	0.0234 (7)	0.0048 (5)	0.0087 (6)	0.0041 (5)
C40	0.0251 (8)	0.0204 (7)	0.0225 (7)	0.0062 (6)	0.0091 (6)	0.0023 (6)
C41	0.0177 (7)	0.0203 (7)	0.0186 (7)	0.0002 (5)	0.0051 (5)	0.0031 (5)
C42	0.0194 (7)	0.0156 (6)	0.0219 (7)	0.0017 (5)	0.0038 (5)	0.0036 (5)
C43	0.0164 (6)	0.0161 (6)	0.0211 (7)	0.0040 (5)	0.0038 (5)	0.0031 (5)
C44	0.0171 (6)	0.0201 (7)	0.0210 (7)	0.0053 (5)	0.0013 (5)	0.0074 (5)
C45	0.0180 (7)	0.0176 (7)	0.0208 (7)	0.0032 (5)	0.0033 (5)	0.0042 (5)
C46	0.0192 (7)	0.0166 (6)	0.0171 (6)	0.0026 (5)	0.0005 (5)	0.0037 (5)
C47	0.0151 (6)	0.0191 (7)	0.0194 (7)	0.0042 (5)	0.0029 (5)	0.0036 (5)
C48	0.0180 (7)	0.0205 (7)	0.0199 (7)	0.0049 (5)	0.0052 (5)	0.0042 (5)
C49	0.0150 (6)	0.0183 (7)	0.0169 (6)	0.0023 (5)	0.0019 (5)	0.0032 (5)
C50	0.0203 (7)	0.0143 (6)	0.0216 (7)	0.0033 (5)	0.0035 (5)	0.0032 (5)
C51	0.0214 (7)	0.0198 (7)	0.0193 (7)	0.0048 (6)	0.0061 (5)	0.0031 (5)
C52	0.0165 (6)	0.0188 (7)	0.0209 (7)	0.0003 (5)	0.0031 (5)	0.0018 (5)
C53	0.0239 (8)	0.0348 (9)	0.0262 (8)	0.0080 (7)	−0.0030 (6)	0.0049 (7)
C54	0.0530 (12)	0.0236 (8)	0.0268 (9)	0.0149 (8)	0.0134 (8)	−0.0011 (7)
C55	0.0458 (11)	0.0329 (10)	0.0303 (9)	0.0063 (8)	0.0239 (8)	0.0037 (8)
Cl1	0.0548 (4)	0.0621 (4)	0.0490 (3)	0.0249 (3)	0.0048 (3)	−0.0132 (3)
C56	0.035 (2)	0.0289 (18)	0.039 (2)	0.0057 (15)	0.0161 (16)	0.0012 (16)

### Geometric parameters (Å, °)

**Table:**

Ru1—C45	1.8834 (16)	C15—C16	1.388 (2)
Ru1—C44	1.9369 (16)	C15—H15A	0.9300
Ru1—C46	1.9402 (16)	C16—C17	1.380 (3)
Ru1—As1	2.43231 (19)	C16—H16A	0.9300
Ru1—Ru2	2.85874 (16)	C17—C18	1.377 (3)
Ru1—Ru3	2.88091 (16)	C17—H17A	0.9300
Ru2—C48	1.8958 (16)	C18—C19	1.397 (2)
Ru2—C47	1.9288 (16)	C18—H18A	0.9300
Ru2—C49	1.9344 (15)	C19—H19A	0.9300
Ru2—As2	2.42827 (18)	C20—C21	1.393 (2)
Ru2—Ru3	2.81795 (16)	C20—C25	1.396 (2)
Ru3—C51	1.8825 (16)	C21—C22	1.393 (2)
Ru3—C52	1.9265 (16)	C21—H21A	0.9300
Ru3—C50	1.9413 (16)	C22—C23	1.385 (3)
Ru3—As3	2.44608 (19)	C22—H22A	0.9300
As1—C7	1.9404 (15)	C23—C24	1.386 (3)
As1—C1	1.9546 (15)	C23—H23A	0.9300
As1—C13	1.9594 (15)	C24—C25	1.391 (2)
As2—C20	1.9309 (15)	C24—H24A	0.9300
As2—C14	1.9380 (15)	C25—H25A	0.9300
As2—C13	1.9562 (15)	C26—C31	1.389 (2)
As3—C26	1.9408 (15)	C26—C27	1.396 (2)
As3—C38	1.9413 (14)	C27—C28	1.384 (2)
As3—C32	1.9450 (15)	C27—H27A	0.9300
O1—C44	1.143 (2)	C28—C29	1.394 (2)
O2—C45	1.1502 (19)	C28—H28A	0.9300
O3—C46	1.1442 (19)	C29—C30	1.395 (2)
O4—C47	1.1458 (19)	C30—C31	1.393 (2)
O5—C48	1.145 (2)	C30—H30A	0.9300
O6—C49	1.1449 (19)	C31—H31A	0.9300
O7—C50	1.1419 (19)	C32—C37	1.382 (2)
O8—C51	1.145 (2)	C32—C33	1.402 (2)
O9—C52	1.150 (2)	C33—C34	1.381 (2)
O10—C29	1.3582 (19)	C33—H33A	0.9300
O10—C53	1.434 (2)	C34—C35	1.394 (2)
O11—C35	1.3639 (19)	C34—H34A	0.9300
O11—C54	1.416 (2)	C35—C36	1.388 (2)
O12—C41	1.3688 (19)	C36—C37	1.396 (2)
O12—C55	1.433 (2)	C36—H36A	0.9300
C1—C6	1.393 (2)	C37—H37A	0.9300
C1—C2	1.397 (2)	C38—C43	1.394 (2)
C2—C3	1.394 (3)	C38—C39	1.398 (2)
C2—H2A	0.9300	C39—C40	1.390 (2)
C3—C4	1.387 (3)	C39—H39A	0.9300
C3—H3A	0.9300	C40—C41	1.390 (2)
C4—C5	1.385 (3)	C40—H40A	0.9300
C4—H4A	0.9300	C41—C42	1.399 (2)
C5—C6	1.396 (2)	C42—C43	1.387 (2)
C5—H5A	0.9300	C42—H42A	0.9300
C6—H6A	0.9300	C43—H43A	0.9300
C7—C8	1.391 (2)	C53—H53A	0.9600
C7—C12	1.391 (2)	C53—H53B	0.9600
C8—C9	1.392 (2)	C53—H53C	0.9600
C8—H8A	0.9300	C54—H54A	0.9600
C9—C10	1.381 (3)	C54—H54B	0.9600
C9—H9A	0.9300	C54—H54C	0.9600
C10—C11	1.382 (3)	C55—H55A	0.9600
C10—H10A	0.9300	C55—H55B	0.9600
C11—C12	1.388 (2)	C55—H55C	0.9600
C11—H11A	0.9300	Cl1—C56	1.687 (4)
C12—H12A	0.9300	Cl1—C56^i^	1.707 (4)
C13—H13A	0.9700	C56—C56^i^	1.682 (8)
C13—H13B	0.9700	C56—Cl1^i^	1.707 (4)
C14—C19	1.384 (2)	C56—H56A	0.9601
C14—C15	1.395 (2)	C56—H56B	0.9599
			
C45—Ru1—C44	92.98 (7)	C18—C17—H17A	120.0
C45—Ru1—C46	91.58 (7)	C16—C17—H17A	120.0
C44—Ru1—C46	170.65 (6)	C17—C18—C19	120.41 (18)
C45—Ru1—As1	97.62 (5)	C17—C18—H18A	119.8
C44—Ru1—As1	92.39 (5)	C19—C18—H18A	119.8
C46—Ru1—As1	95.09 (4)	C14—C19—C18	119.64 (17)
C45—Ru1—Ru2	167.46 (5)	C14—C19—H19A	120.2
C44—Ru1—Ru2	93.41 (4)	C18—C19—H19A	120.2
C46—Ru1—Ru2	80.64 (4)	C21—C20—C25	119.72 (14)
As1—Ru1—Ru2	92.883 (6)	C21—C20—As2	118.52 (11)
C45—Ru1—Ru3	112.52 (5)	C25—C20—As2	121.64 (12)
C44—Ru1—Ru3	76.37 (5)	C22—C21—C20	120.06 (15)
C46—Ru1—Ru3	94.34 (4)	C22—C21—H21A	120.0
As1—Ru1—Ru3	148.088 (6)	C20—C21—H21A	120.0
Ru2—Ru1—Ru3	58.806 (4)	C23—C22—C21	120.08 (16)
C48—Ru2—C47	90.02 (7)	C23—C22—H22A	120.0
C48—Ru2—C49	92.26 (6)	C21—C22—H22A	120.0
C47—Ru2—C49	173.16 (6)	C22—C23—C24	120.02 (15)
C48—Ru2—As2	103.42 (5)	C22—C23—H23A	120.0
C47—Ru2—As2	95.92 (5)	C24—C23—H23A	120.0
C49—Ru2—As2	89.82 (4)	C23—C24—C25	120.41 (16)
C48—Ru2—Ru3	106.08 (5)	C23—C24—H24A	119.8
C47—Ru2—Ru3	77.30 (4)	C25—C24—H24A	119.8
C49—Ru2—Ru3	95.87 (4)	C24—C25—C20	119.71 (16)
As2—Ru2—Ru3	149.666 (6)	C24—C25—H25A	120.1
C48—Ru2—Ru1	164.14 (5)	C20—C25—H25A	120.1
C47—Ru2—Ru1	95.47 (4)	C31—C26—C27	118.82 (14)
C49—Ru2—Ru1	80.70 (4)	C31—C26—As3	123.03 (11)
As2—Ru2—Ru1	90.831 (6)	C27—C26—As3	117.42 (11)
Ru3—Ru2—Ru1	60.990 (4)	C28—C27—C26	120.76 (14)
C51—Ru3—C52	93.67 (7)	C28—C27—H27A	119.6
C51—Ru3—C50	93.66 (7)	C26—C27—H27A	119.6
C52—Ru3—C50	172.38 (7)	C27—C28—C29	119.88 (15)
C51—Ru3—As3	98.10 (5)	C27—C28—H28A	120.1
C52—Ru3—As3	91.69 (5)	C29—C28—H28A	120.1
C50—Ru3—As3	89.31 (5)	O10—C29—C28	115.59 (14)
C51—Ru3—Ru2	91.66 (5)	O10—C29—C30	124.26 (15)
C52—Ru3—Ru2	97.43 (4)	C28—C29—C30	120.15 (14)
C50—Ru3—Ru2	80.30 (5)	C31—C30—C29	119.14 (14)
As3—Ru3—Ru2	166.195 (7)	C31—C30—H30A	120.4
C51—Ru3—Ru1	148.00 (5)	C29—C30—H30A	120.4
C52—Ru3—Ru1	76.72 (5)	C26—C31—C30	121.20 (14)
C50—Ru3—Ru1	95.92 (4)	C26—C31—H31A	119.4
As3—Ru3—Ru1	112.451 (6)	C30—C31—H31A	119.4
Ru2—Ru3—Ru1	60.204 (4)	C37—C32—C33	118.66 (14)
C7—As1—C1	98.44 (7)	C37—C32—As3	121.90 (12)
C7—As1—C13	101.20 (6)	C33—C32—As3	119.40 (11)
C1—As1—C13	103.56 (6)	C34—C33—C32	120.45 (15)
C7—As1—Ru1	116.04 (4)	C34—C33—H33A	119.8
C1—As1—Ru1	119.41 (5)	C32—C33—H33A	119.8
C13—As1—Ru1	115.32 (4)	C33—C34—C35	120.18 (16)
C20—As2—C14	103.87 (6)	C33—C34—H34A	119.9
C20—As2—C13	103.95 (7)	C35—C34—H34A	119.9
C14—As2—C13	99.26 (6)	O11—C35—C36	124.82 (16)
C20—As2—Ru2	118.46 (5)	O11—C35—C34	115.04 (15)
C14—As2—Ru2	118.68 (4)	C36—C35—C34	120.13 (15)
C13—As2—Ru2	110.06 (4)	C35—C36—C37	119.06 (15)
C26—As3—C38	102.24 (6)	C35—C36—H36A	120.5
C26—As3—C32	97.67 (6)	C37—C36—H36A	120.5
C38—As3—C32	101.39 (6)	C32—C37—C36	121.52 (15)
C26—As3—Ru3	122.47 (4)	C32—C37—H37A	119.2
C38—As3—Ru3	114.66 (4)	C36—C37—H37A	119.2
C32—As3—Ru3	115.14 (5)	C43—C38—C39	118.62 (14)
C29—O10—C53	117.47 (14)	C43—C38—As3	120.13 (11)
C35—O11—C54	117.28 (15)	C39—C38—As3	121.17 (11)
C41—O12—C55	116.78 (14)	C40—C39—C38	121.08 (14)
C6—C1—C2	119.99 (15)	C40—C39—H39A	119.5
C6—C1—As1	120.64 (12)	C38—C39—H39A	119.5
C2—C1—As1	119.32 (13)	C41—C40—C39	119.66 (15)
C3—C2—C1	119.77 (18)	C41—C40—H40A	120.2
C3—C2—H2A	120.1	C39—C40—H40A	120.2
C1—C2—H2A	120.1	O12—C41—C40	124.78 (15)
C4—C3—C2	120.10 (18)	O12—C41—C42	115.45 (14)
C4—C3—H3A	120.0	C40—C41—C42	119.76 (14)
C2—C3—H3A	120.0	C43—C42—C41	120.07 (14)
C5—C4—C3	120.27 (17)	C43—C42—H42A	120.0
C5—C4—H4A	119.9	C41—C42—H42A	120.0
C3—C4—H4A	119.9	C42—C43—C38	120.71 (14)
C4—C5—C6	120.12 (18)	C42—C43—H43A	119.6
C4—C5—H5A	119.9	C38—C43—H43A	119.6
C6—C5—H5A	119.9	O1—C44—Ru1	173.41 (14)
C1—C6—C5	119.75 (16)	O2—C45—Ru1	175.91 (14)
C1—C6—H6A	120.1	O3—C46—Ru1	175.41 (13)
C5—C6—H6A	120.1	O4—C47—Ru2	174.12 (14)
C8—C7—C12	119.57 (15)	O5—C48—Ru2	178.63 (14)
C8—C7—As1	123.70 (12)	O6—C49—Ru2	174.76 (13)
C12—C7—As1	116.71 (12)	O7—C50—Ru3	174.62 (14)
C7—C8—C9	120.07 (16)	O8—C51—Ru3	174.61 (15)
C7—C8—H8A	120.0	O9—C52—Ru3	172.70 (14)
C9—C8—H8A	120.0	O10—C53—H53A	109.5
C10—C9—C8	119.99 (17)	O10—C53—H53B	109.5
C10—C9—H9A	120.0	H53A—C53—H53B	109.5
C8—C9—H9A	120.0	O10—C53—H53C	109.5
C9—C10—C11	120.13 (17)	H53A—C53—H53C	109.5
C9—C10—H10A	119.9	H53B—C53—H53C	109.5
C11—C10—H10A	119.9	O11—C54—H54A	109.5
C10—C11—C12	120.32 (17)	O11—C54—H54B	109.5
C10—C11—H11A	119.8	H54A—C54—H54B	109.5
C12—C11—H11A	119.8	O11—C54—H54C	109.5
C11—C12—C7	119.92 (16)	H54A—C54—H54C	109.5
C11—C12—H12A	120.0	H54B—C54—H54C	109.5
C7—C12—H12A	120.0	O12—C55—H55A	109.5
As2—C13—As1	111.05 (7)	O12—C55—H55B	109.5
As2—C13—H13A	109.4	H55A—C55—H55B	109.5
As1—C13—H13A	109.4	O12—C55—H55C	109.5
As2—C13—H13B	109.4	H55A—C55—H55C	109.5
As1—C13—H13B	109.4	H55B—C55—H55C	109.5
H13A—C13—H13B	108.0	C56—Cl1—C56^i^	59.4 (2)
C19—C14—C15	119.75 (14)	C56^i^—C56—Cl1	60.9 (3)
C19—C14—As2	123.05 (12)	C56^i^—C56—Cl1^i^	59.7 (3)
C15—C14—As2	117.19 (11)	Cl1—C56—Cl1^i^	120.6 (2)
C16—C15—C14	119.96 (16)	C56^i^—C56—H56A	126.6
C16—C15—H15A	120.0	Cl1—C56—H56A	107.2
C14—C15—H15A	120.0	Cl1^i^—C56—H56A	107.2
C17—C16—C15	120.17 (17)	C56^i^—C56—H56B	126.6
C17—C16—H16A	119.9	Cl1—C56—H56B	107.2
C15—C16—H16A	119.9	Cl1^i^—C56—H56B	107.2
C18—C17—C16	120.05 (16)	H56A—C56—H56B	106.8
			
C45—Ru1—Ru2—C48	−86.2 (3)	C7—As1—C1—C6	−137.28 (14)
C44—Ru1—Ru2—C48	34.25 (18)	C13—As1—C1—C6	118.97 (14)
C46—Ru1—Ru2—C48	−138.49 (18)	Ru1—As1—C1—C6	−10.90 (15)
As1—Ru1—Ru2—C48	126.82 (18)	C7—As1—C1—C2	40.08 (14)
Ru3—Ru1—Ru2—C48	−37.56 (18)	C13—As1—C1—C2	−63.68 (14)
C45—Ru1—Ru2—C47	23.5 (2)	Ru1—As1—C1—C2	166.45 (12)
C44—Ru1—Ru2—C47	144.02 (7)	C6—C1—C2—C3	0.0 (3)
C46—Ru1—Ru2—C47	−28.72 (6)	As1—C1—C2—C3	−177.41 (15)
As1—Ru1—Ru2—C47	−123.41 (5)	C1—C2—C3—C4	−0.1 (3)
Ru3—Ru1—Ru2—C47	72.21 (5)	C2—C3—C4—C5	0.3 (3)
C45—Ru1—Ru2—C49	−150.8 (2)	C3—C4—C5—C6	−0.2 (3)
C44—Ru1—Ru2—C49	−30.26 (7)	C2—C1—C6—C5	0.0 (3)
C46—Ru1—Ru2—C49	157.00 (6)	As1—C1—C6—C5	177.38 (14)
As1—Ru1—Ru2—C49	62.30 (4)	C4—C5—C6—C1	0.1 (3)
Ru3—Ru1—Ru2—C49	−102.08 (4)	C1—As1—C7—C8	−102.24 (14)
C45—Ru1—Ru2—As2	119.5 (2)	C13—As1—C7—C8	3.48 (15)
C44—Ru1—Ru2—As2	−119.95 (5)	Ru1—As1—C7—C8	129.08 (13)
C46—Ru1—Ru2—As2	67.31 (5)	C1—As1—C7—C12	76.16 (13)
As1—Ru1—Ru2—As2	−27.382 (7)	C13—As1—C7—C12	−178.11 (13)
Ru3—Ru1—Ru2—As2	168.231 (6)	Ru1—As1—C7—C12	−52.52 (13)
C45—Ru1—Ru2—Ru3	−48.7 (2)	C12—C7—C8—C9	0.7 (3)
C44—Ru1—Ru2—Ru3	71.82 (5)	As1—C7—C8—C9	179.07 (14)
C46—Ru1—Ru2—Ru3	−100.92 (5)	C7—C8—C9—C10	−0.7 (3)
As1—Ru1—Ru2—Ru3	164.387 (6)	C8—C9—C10—C11	0.1 (3)
C48—Ru2—Ru3—C51	−26.04 (7)	C9—C10—C11—C12	0.5 (3)
C47—Ru2—Ru3—C51	60.26 (7)	C10—C11—C12—C7	−0.5 (3)
C49—Ru2—Ru3—C51	−120.10 (6)	C8—C7—C12—C11	−0.1 (3)
As2—Ru2—Ru3—C51	140.13 (5)	As1—C7—C12—C11	−178.59 (14)
Ru1—Ru2—Ru3—C51	163.95 (5)	C20—As2—C13—As1	83.43 (8)
C48—Ru2—Ru3—C52	−119.96 (7)	C14—As2—C13—As1	−169.67 (8)
C47—Ru2—Ru3—C52	−33.67 (7)	Ru2—As2—C13—As1	−44.41 (8)
C49—Ru2—Ru3—C52	145.97 (7)	C7—As1—C13—As2	144.77 (8)
As2—Ru2—Ru3—C52	46.21 (5)	C1—As1—C13—As2	−113.60 (8)
Ru1—Ru2—Ru3—C52	70.03 (5)	Ru1—As1—C13—As2	18.69 (9)
C48—Ru2—Ru3—C50	67.39 (7)	C20—As2—C14—C19	2.79 (16)
C47—Ru2—Ru3—C50	153.69 (7)	C13—As2—C14—C19	−104.18 (15)
C49—Ru2—Ru3—C50	−26.67 (6)	Ru2—As2—C14—C19	136.78 (14)
As2—Ru2—Ru3—C50	−126.44 (5)	C20—As2—C14—C15	−177.63 (12)
Ru1—Ru2—Ru3—C50	−102.62 (5)	C13—As2—C14—C15	75.40 (13)
C48—Ru2—Ru3—As3	109.10 (6)	Ru2—As2—C14—C15	−43.63 (14)
C47—Ru2—Ru3—As3	−164.60 (5)	C19—C14—C15—C16	0.0 (3)
C49—Ru2—Ru3—As3	15.04 (5)	As2—C14—C15—C16	−179.62 (13)
As2—Ru2—Ru3—As3	−84.73 (3)	C14—C15—C16—C17	−0.7 (3)
Ru1—Ru2—Ru3—As3	−60.91 (3)	C15—C16—C17—C18	1.3 (3)
C48—Ru2—Ru3—Ru1	170.01 (5)	C16—C17—C18—C19	−1.2 (3)
C47—Ru2—Ru3—Ru1	−103.69 (5)	C15—C14—C19—C18	0.2 (3)
C49—Ru2—Ru3—Ru1	75.95 (4)	As2—C14—C19—C18	179.75 (16)
As2—Ru2—Ru3—Ru1	−23.816 (12)	C17—C18—C19—C14	0.4 (3)
C45—Ru1—Ru3—C51	138.41 (10)	C14—As2—C20—C21	79.70 (13)
C44—Ru1—Ru3—C51	−134.04 (10)	C13—As2—C20—C21	−176.88 (12)
C46—Ru1—Ru3—C51	44.88 (10)	Ru2—As2—C20—C21	−54.42 (13)
As1—Ru1—Ru3—C51	−61.99 (9)	C14—As2—C20—C25	−104.31 (13)
Ru2—Ru1—Ru3—C51	−31.43 (9)	C13—As2—C20—C25	−0.89 (14)
C45—Ru1—Ru3—C52	63.08 (7)	Ru2—As2—C20—C25	121.56 (12)
C44—Ru1—Ru3—C52	150.64 (7)	C25—C20—C21—C22	−0.8 (2)
C46—Ru1—Ru3—C52	−30.44 (7)	As2—C20—C21—C22	175.23 (12)
As1—Ru1—Ru3—C52	−137.32 (5)	C20—C21—C22—C23	0.5 (2)
Ru2—Ru1—Ru3—C52	−106.75 (5)	C21—C22—C23—C24	0.1 (3)
C45—Ru1—Ru3—C50	−114.91 (7)	C22—C23—C24—C25	−0.4 (3)
C44—Ru1—Ru3—C50	−27.36 (7)	C23—C24—C25—C20	0.1 (3)
C46—Ru1—Ru3—C50	151.56 (6)	C21—C20—C25—C24	0.5 (2)
As1—Ru1—Ru3—C50	44.69 (5)	As2—C20—C25—C24	−175.41 (13)
Ru2—Ru1—Ru3—C50	75.25 (5)	C38—As3—C26—C31	−14.08 (14)
C45—Ru1—Ru3—As3	−23.20 (5)	C32—As3—C26—C31	89.40 (13)
C44—Ru1—Ru3—As3	64.35 (5)	Ru3—As3—C26—C31	−144.18 (11)
C46—Ru1—Ru3—As3	−116.73 (4)	C38—As3—C26—C27	175.92 (12)
As1—Ru1—Ru3—As3	136.396 (12)	C32—As3—C26—C27	−80.60 (12)
Ru2—Ru1—Ru3—As3	166.961 (7)	Ru3—As3—C26—C27	45.83 (13)
C45—Ru1—Ru3—Ru2	169.84 (5)	C31—C26—C27—C28	−2.1 (2)
C44—Ru1—Ru3—Ru2	−102.61 (5)	As3—C26—C27—C28	168.36 (12)
C46—Ru1—Ru3—Ru2	76.31 (4)	C26—C27—C28—C29	0.3 (2)
As1—Ru1—Ru3—Ru2	−30.564 (12)	C53—O10—C29—C28	178.52 (15)
C45—Ru1—As1—C7	78.69 (7)	C53—O10—C29—C30	−1.3 (2)
C44—Ru1—As1—C7	−14.63 (7)	C27—C28—C29—O10	−177.84 (15)
C46—Ru1—As1—C7	170.98 (7)	C27—C28—C29—C30	2.0 (2)
Ru2—Ru1—As1—C7	−108.17 (5)	O10—C29—C30—C31	177.28 (15)
Ru3—Ru1—As1—C7	−82.35 (5)	C28—C29—C30—C31	−2.6 (2)
C45—Ru1—As1—C1	−38.88 (7)	C27—C26—C31—C30	1.5 (2)
C44—Ru1—As1—C1	−132.21 (7)	As3—C26—C31—C30	−168.36 (12)
C46—Ru1—As1—C1	53.41 (7)	C29—C30—C31—C26	0.8 (2)
Ru2—Ru1—As1—C1	134.26 (5)	C26—As3—C32—C37	141.49 (13)
Ru3—Ru1—As1—C1	160.07 (5)	C38—As3—C32—C37	−114.31 (13)
C45—Ru1—As1—C13	−163.25 (7)	Ru3—As3—C32—C37	10.07 (15)
C44—Ru1—As1—C13	103.43 (7)	C26—As3—C32—C33	−36.08 (13)
C46—Ru1—As1—C13	−70.96 (7)	C38—As3—C32—C33	68.13 (13)
Ru2—Ru1—As1—C13	9.89 (5)	Ru3—As3—C32—C33	−167.49 (11)
Ru3—Ru1—As1—C13	35.71 (5)	C37—C32—C33—C34	0.5 (2)
C48—Ru2—As2—C20	111.52 (7)	As3—C32—C33—C34	178.18 (13)
C47—Ru2—As2—C20	20.08 (7)	C32—C33—C34—C35	0.4 (3)
C49—Ru2—As2—C20	−156.20 (7)	C54—O11—C35—C36	−4.1 (2)
Ru3—Ru2—As2—C20	−54.83 (5)	C54—O11—C35—C34	177.41 (15)
Ru1—Ru2—As2—C20	−75.51 (5)	C33—C34—C35—O11	177.55 (15)
C48—Ru2—As2—C14	−15.89 (7)	C33—C34—C35—C36	−1.0 (3)
C47—Ru2—As2—C14	−107.32 (7)	O11—C35—C36—C37	−177.68 (16)
C49—Ru2—As2—C14	76.40 (7)	C34—C35—C36—C37	0.8 (2)
Ru3—Ru2—As2—C14	177.77 (5)	C33—C32—C37—C36	−0.8 (2)
Ru1—Ru2—As2—C14	157.09 (5)	As3—C32—C37—C36	−178.41 (13)
C48—Ru2—As2—C13	−129.15 (7)	C35—C36—C37—C32	0.2 (3)
C47—Ru2—As2—C13	139.41 (7)	C26—As3—C38—C43	127.25 (12)
C49—Ru2—As2—C13	−36.87 (7)	C32—As3—C38—C43	26.71 (13)
Ru3—Ru2—As2—C13	64.51 (5)	Ru3—As3—C38—C43	−97.99 (12)
Ru1—Ru2—As2—C13	43.83 (5)	C26—As3—C38—C39	−56.06 (14)
C51—Ru3—As3—C26	−166.95 (7)	C32—As3—C38—C39	−156.61 (13)
C52—Ru3—As3—C26	−73.00 (7)	Ru3—As3—C38—C39	78.69 (13)
C50—Ru3—As3—C26	99.45 (7)	C43—C38—C39—C40	−2.5 (2)
Ru2—Ru3—As3—C26	58.46 (6)	As3—C38—C39—C40	−179.28 (13)
Ru1—Ru3—As3—C26	3.32 (5)	C38—C39—C40—C41	2.1 (3)
C51—Ru3—As3—C38	68.39 (7)	C55—O12—C41—C40	1.5 (2)
C52—Ru3—As3—C38	162.35 (7)	C55—O12—C41—C42	−179.25 (16)
C50—Ru3—As3—C38	−25.20 (7)	C39—C40—C41—O12	179.91 (16)
Ru2—Ru3—As3—C38	−66.19 (6)	C39—C40—C41—C42	0.6 (2)
Ru1—Ru3—As3—C38	−121.33 (5)	O12—C41—C42—C43	177.84 (14)
C51—Ru3—As3—C32	−48.70 (7)	C40—C41—C42—C43	−2.8 (2)
C52—Ru3—As3—C32	45.25 (7)	C41—C42—C43—C38	2.3 (2)
C50—Ru3—As3—C32	−142.30 (7)	C39—C38—C43—C42	0.3 (2)
Ru2—Ru3—As3—C32	176.71 (5)	As3—C38—C43—C42	177.09 (12)
Ru1—Ru3—As3—C32	121.57 (5)	C56^i^—Cl1—C56—Cl1^i^	−0.003 (2)

Symmetry codes: (i) −*x*+1, −*y*+1, −*z*+1.

### Hydrogen-bond geometry (Å, °)

**Table:**

Cg1 Cg2 and Cg3 are the centroids of the C38–C43, C26–C31 and C32–C37 benzene rings, respectively.

**Table:**

*D*—H···*A*	*D*—H	H···*A*	*D*···*A*	*D*—H···*A*
C17—H17A···Cg1^ii^	0.93	2.92	3.813 (2)	161
C22—H22A···Cg2^iii^	0.93	2.89	3.5863 (19)	133
C54—H54C···Cg3^iv^	0.96	2.87	3.710 (2)	147

Symmetry codes: (ii) −*x*+2, −*y*+1, −*z*+1; (iii) *x*+1, *y*, *z*; (iv) −*x*+1, −*y*, −*z*.
